# Explaining the Relationship Between Post-Critical Beliefs and Sense of Coherence in Polish Young, Middle, and Late Adults

**DOI:** 10.1007/s10943-013-9680-7

**Published:** 2013-02-01

**Authors:** Beata Zarzycka, Elżbieta Rydz

**Affiliations:** 1 Department of Social Psychology and Psychology of Religion, The John Paul II Catholic University of Lublin, Al. Racławickie 14, 20-950 Lublin, Poland; 2 Department of Developmental Psychology, The John Paul II Catholic University of Lublin, Lublin, Poland

**Keywords:** Sense of coherence, Post-critical beliefs, Religiosity

## Abstract

The subject of the presented research is the analysis of relations between Post-Critical Belief and Sense of Coherence in women and men in early, middle, and late adulthood. Six hundred and thirty-six individuals participated in the research, 332 women and 304 men, at the age of 18–79 years. We applied the Post-Critical Belief scale by Hutsebaut (J Empir Theol 9:48–66, 1996) and the Sense of Coherence scale (SOC-29) by Antonovsky (Soc Sci Med 36:725–733, 1993). The results suggest that the salutogenic function of religiosity is related to age and gender—in women, it is most strongly marked in late, and in men, in middle adulthood

## Introduction

Religion is among the most important cultural factors which give structure and meaning to human behaviors and experiences. Surveys of the US population have established that religion holds a central place in the lives of many Americans. In light of the research conducted by the Bertesmann Foundation in 2008, 68 % of Americans declared high importance of religious beliefs and prayer in their life (Joas 2009). Previous studies revealed even higher indicators. According to the data by Gallup and Lindsay (1981), almost 75 % of the American society hold the opinion that their attitude to live is rooted in their religious beliefs. Other studies (see McNichol 1996; Tagay et al. 2006) indicated that 79 % of Americans perceive their own religious and spiritual activity as the source of consolation in the face of illness. A considerable group of interviewed individuals (56 %) believed that religion helped them recover, and 63 % suggested that doctors should talk with patients about religious issues. In Poland, as many as 95 % of the society declare their affiliation with Catholicism but only 41 % consider themselves very religious and the same percentage refers to the individuals who believe that religion helps people cope with illness (Zarzycka 2009).

A considerable amount of empirical data indicate that religious commitment may play a beneficial role in preventing mental and physical illness, improving the way people cope with mental and physical illness, and facilitating recovery from illness and distress (Acklin et al. 1983; Ryan et al. 1993; Mueller et al. 2001; Tagay et al. 2006). The majority of the nearly 350 studies of physical health and 850 studies of mental health that have used religious and spiritual variables indicated that religious involvement and spirituality were associated with better health outcomes (Mueller et al. 2001; Tagay et al. 2006). The frequency of participation in religious services correlated with lower mortality rate, low blood pressure, and low level of depression and somatic symptoms (Schumacher et al. 2000). Researchers noted also positive relationships of religious commitment with well-being and existential coherence (Ellison 1991; Unterrainer et al. 2010), negative relationships of religious coping with neuroticism, anxiety and positive with extraversion (Saraglou 2002; Piedmont 2005; Śliwak and Zarzycka 2012). Moreover, intrinsic religiosity correlated positively with satisfaction with life (Zwingmann 1991), personal adaptation (Bergin et al. 1987; Koenig et al. 1988; Watson et al. 1994), self-esteem (Nelson 1990; Ryan et al. 1993), internal locus of control (Kahoe 1974; Jackson and Coursey 1988), and purpose in life (Crandall and Rasmussen 1975).

Despite the fact that numerous authors assert that there is a substantial empirical support for the idea that religious commitment promotes health (Koenig et al. 2001), there are researchers who believe that the support for the beneficial role of religiosity is weak and unconvincing. This is because many data come from studies which arouse methodological reservations or from studies which lack clarity and precision (Sloan and Bagiella 2002; Tagay et al. 2006). Moreover, many researchers describe religiosity as a unidimensional construct. Even if multidimensional religiosity concepts are presented, they are based on distinctions such as intrinsic versus extrinsic religiosity (Allport and Ross 1967), criticized on both conceptual and psychometric grounds (Kirkpatrick and Hood 1990). However, there is a recently developed idea of the Post-Critical Belief scale (PCBS) (Hutsebaut 1996, 1997; Duriez et al. 2000), which operationalizes Wulff’s (1991, 1999) model of attitudes to religion, and thus has opened new perspectives for studying religiosity–health’s outcomes relations. Wulff suggested four potential religious attitudes: Literal Affirmation, Literal Disaffirmation, Reductive Interpretation, and Restorative Interpretation. In our study, we investigate the relations of Wulff’s (1991, 1999) approaches to religion and Sense of Coherence (SOC). First, we will present the theoretical framework of Wulff’s conceptualization (1991, 1999) and the PCBS (Fontaine et al. 2003), which operationalizes Wulff’s concept. Next, we will introduce the concept of SOC by Antonovsky (1993). Finally, we will make predictions regarding the relations between attitudes to religion, on the one hand, and SOC, on the other hand.

### Wullf’s Conceptualization of Attitudes to Religion

**Fig. 1:**
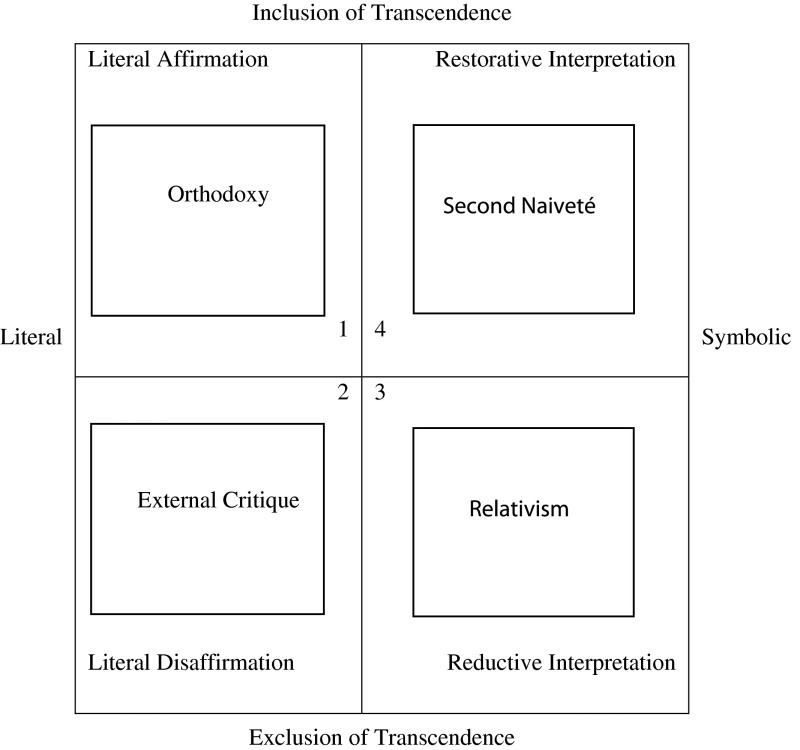
Integration of the three Post-Critical Belief subscales in Wulff’s (1991, 1999) theoretical model according to Hutsebaut (1996) (see Fontaine et al. 2003)

David Wulff (1991, 1999) demonstrated a new and interesting perspective on religion in a secularized sociocultural context. Wulff (1991, 1999) brought forward the argument that the various possible attitudes toward religion can be positioned in a two-dimensional space (see Fig. 1). The vertical axis in this space shows the degree to which the objects of religious interest are granted participation in a transcendent reality (Exclusion vs. Inclusion of Transcendence). The horizontal axis indicates whether individuals interpret religion literally or symbolically (Literal vs Symbolic). In this way, the two dimensions define four quadrants, each reflecting a potential religious attitude (see Fig. 1):

- Literal Affirmation—this is a position which is in particular included in religious fundamentalism. Wulff (1991, 1999) suggested that individuals can sustain this position only if she or he accepts the validity of the conservative view. Literal believers tend to have higher scores on measures of prejudice and lower on cognitive development. Typically, they are rigid and low in their ability to adapt. They interpret the religious realm and religious doctrines literally and accept their existence.
- Literal Disaffirmation—this is a position in which the individual does not accept the religious realm. Next, there is no symbolic meaning of the religious language—it is understood only literally. In contrast to Literal Affirmation, individuals who demonstrate Literal Disaffirmation do not accept concepts presented as religious doctrines or dogmas. The sole acceptable absolute concepts refer to scientific methods and rational and formal principles of knowledge.
- Reductive Interpretation—this is a position in which an individual rejects the religious realm. However, he or she acknowledges a privileged perspective on the hidden meaning of religion’s myths and rituals and does not reject symbolic functions of religion. This position stems from the work of Ricoeur (1970), who proposed that a reductive interpretation is necessary in modern hermeneutics to shift from religious symbols, the excrescence of idolatry and illusion. Wulff (1991, 1999) indicated that people representing Reductive Interpretation are complex, socially sensitive, insightful, relatively unprejudiced, and original.
- Restorative Interpretation—this is a position in which the individual affirms the religious realm. However, he or she tries to encompass and transcend all possible Reductive Interpretations in order to find the symbolic meaning of the religious language. Again, this position stems from the work of Ricoeur (1970), who proposed that Restorative Interpretation is necessary in modern hermeneutics to make it possible for the object of suspicion to be restored to an object of understanding and faith. On the basis of this posture, Ricoeur introduced the concept of Second Naivetè. Wulff (1991, 1999) suggested that it is quite difficult to characterize individuals who occupy this position, because researchers have largely neglected this area in empirical research until recently. Nevertheless, Fowler (1980) encompassed this position in his fifth stage (conjunctive faith) of faith development.

Inspired by Wulff (1991, 1999), Hutsebaut and his colleagues (Hutsebaut 1996; Fontaine et al. 2003) constructed the PCBS to measure the four religious attitudes. The PCBS consists of four subscales: Orthodoxy is the measure of Literal Affirmation, External Critique measures Literal Disaffirmation; Relativism, Symbolical Disaffirmation; and Second Naiveté, Symbolical Affirmation (Fontaine et al. 2003).

### Sense of Coherence

In the discussion about health and disease, Antonovsky (1979, 2005) promoted a salutogenic view as a counterbalance to the pathogenic view. In his salutogenic model, Antonovsky defines health as a continuum between the two poles of disease and ease. He indicated that an individual’s degree of SOC in life influences his or her position on this continuum and the ability to recover from illness. According to Antonovsky (1979, 2005), SOC is a disposition-type quality or state that serves to promote health and well-being. Antonovsky (1993) introduced SOC in his attempt to understand the conditions determining the damaging result of stress. He described SOC as a global approach to life or an underlying personality characteristic that expresses the extent to which one has a pervasive, enduring though dynamic feeling of confidence that (1) the stimuli deriving from one’s internal and external environments in the course of living are structured, predictable, and explicable (Comprehensibility); (2) the resources are available to one to meet the demands posed by these stimuli (Manageability); and (3) these demands are challenges, worthy of investment and engagement (Meaningfulness). According to Antonovsky (1993), the “resistance resources,” which help individuals experience stress as less threatening, cope with it more effectively, and be less likely to experience stress-related illness, are the beliefs that the world is meaningful, predictable, and manageable. Moreover, Antonovsky (1979, 2005) suggested that the belief systems of cultures and the social institutions in which people participate help them develop SOC. Several researches have suggested that religions typically provide their members with a worldview; this worldview would often seem to meet Antonovsky’s concept of SOC (George et al. 2002). The authors of numerous studies demonstrated that SOC correlated strongly with mental health, well-being, and general satisfaction with life. Thus, SOC seems to serve as a health-promoting resource, strengthening resilience, and developing a positive subjective state of health (Unterrainer et al. 2010). Accordingly, studies confirmed that SOC correlates negatively with stress, and positively with positive coping with daily stressors and maintaining good physical and psychological health (Antonovsky 1993; Eriksson and Lindström 2006; Arévalo et al. 2008). Researchers observed these dependencies regardless of age, gender, ethnic origin, or nationality. Numerous authors believe that they are an empirical confirmation of the thesis that SOC promotes health (Tagay et al. 2006).

### Research Problem

We will explore relationships between Wulff’s approaches to religiosity and SOC. Antonovsky (1993) suggested that systems of sociocultural beliefs may be important predictors of the SOC and that other religious traditions may also have the function of such systems (Berger 1967; Pargament 1990). Tagay et al. (2006) initiated studies in which they analyzes, for example, relations between religiosity and SOC. They applied a shorter version of the SOC scale (SOC-13) and two items for the measure of religiosity (To what extent are you religious? How important is your religion for your life?). The short measure of religiosity may be the reason why they did not observe any statistically significant relations between religiosity and the SOC. The authors also indicate this by saying: “(…) testing the buffering effect of religiosity on mental health, a religiosity scale might be needed. The two religiosity questions used for this paper do not cover the broad dimensions of religiosity” (p. 170). Tagay et al. (2006) suggested further investigation of the complex nature of religion and its effect on psychosocial outcomes. Evidence supports the idea that a religious framework can play the role of a generic mental model that influences appraisals and affects well-being. This is why we decided to apply a complex religiosity description, that is, the Wulff’s model.

Yet, there is ample empirical data which support the opinion that the function of religiosity varies, depending on the specifics of the sample, gender, and the age of research participants (Simpson et al. 2008). First, in their studies of religion and health, researchers employed a wide range of sampling strategies, ranging from convenience samples to representative samples of both particular geographical areas and the United States as a whole. Second, over 50 % of the studies that address the relationship between religion and health are based on samples of older adults (i.e. age 60–65 and older). In one sense, the generalizability of the research base is limited by the preponderance of studies of older people (George et al. 2002). Therefore, deciding to investigate a non-clinical sample, we extended the age range of the participants to the whole adulthood period—from the early (18–30 years of age), through the middle (31–50), till the late adulthood (51–79).

We measured the correlation between the four approaches to religion and SOC. Bearing in mind that Hutsebaut’s model is relatively new and not extensively researched, formulating hypotheses is a rather tentative business, we nevertheless tried to make at least some predictions with regard to the relationship of Wulff`s approaches with religion and SOC:

1. Taking into account the fact that multiple research results indicated a positive function of religiosity in coping with illness and positive correlations of religiosity with health indicators (Mueller et al. 2001; Tagay et al. 2006), we hypothesize that the PCBS measures which describe the Inclusion of Transcendence (Orthodoxy and Second Naiveté) should also correlate positively with SOC. And the PCBS measures which describe the Exclusion of Transcendence (External Critique and Relativism) should correlate negatively with SOC.
2. As numerous researchers accept the considerable positive function of religiosity in the development of the sense of life (Ardelt 2003; Krause 2003; Homan and Boyatzis 2010), we hypothesize that Meaningfulness will have stronger correlations with Orthodoxy and Second Naiveté than the other two SOC subcomponents (Comprehensibility and Manageability).
3. We treat the assumption about the diversification of relations between the dimensions of the PCBS and the SOC-29, depending on age and gender, as explorative in nature, because neither existing research nor theoretical assumption allowed for formulating detailed hypotheses predicting the directions of potential correlations.

## Method

### Participants

The demographic details of the participants are given in Table 1. We obtained reanalyzed data in non-clinical samples (general population). The total sample consisted of 636 participants, 332 women (52.2 %) and 304 men (47.8 %), who ranged in age from 18 to 76 years. The mean age of all participants was 41.70 years (SD = 16.78). Six hundred and fifteen (96.7 %) participants reported their religious affiliation as Catholicism. In terms of education, 50 individuals had elementary, 376 secondary, and 210 higher education. We divided the participants into three age groups: early, middle, and late adulthood. There were 220 early adults, 115 women, and 105 men, aged between 18 and 30. Middle adulthood was represented by 203 individuals, 103 women and 100 men, aged between 31 and 50. Finally, there were 213 participants in the group of late adults, 114 women and 99 men, aged between 51 and 79 (see Table 1).

**Table 1:** Demographic characteristics

Groups	Group						Education						Age	
	Total		Women		Men		Elementary		Secondary		Higher			
	*N*	%	*N*	%	*N*	%	*N*	%	*N*	%	*N*	%	*M*	SD
18–30	220	34.6	115	34.6	105	34.5	10	4.5	147	66.8	63	28.6	22.02	2.74
31–50	203	31.9	103	31.0	100	32.9	7	3.4	105	51.7	91	44.8	42.93	5.12
51–79	213	33.5	114	34.3	99	32.6	33	15.5	124	58.2	56	26.3	60.86	6.23
Total	636	100.0	332	100.0	304	100.0	220	100.0	203	100.0	213	100.0	41.70	16.78

### Measures

#### Participants completed the PCBS (33 items) and the SOC scale (SOC-29) (29 items)

The PCBS consists of four subscales: Orthodoxy (8 items), External Critique (9 items), Relativism (8 items), and Second Naiveté (8 items). All items were scored on a 7-point Likert scale. In our sample, estimates of internal consistency were *α* = 0.71 for Orthodoxy (*M* = 4.38; SD = 1.12), *α* = 0.87 for External Critique (*M* = 3.15; SD = 0.99), *α* = 0.72 for Relativism (*M* = 4.03; SD = 0.96), and *α* = 0.72 for Second Naiveté (*M* = 4.93; SD = 0.78). The authors of Polish adaptation are Bartczuk et al. (2011).

The SOC-29 is based on the concept of salutogenesis by Antonovsky (1993). He introduced his concept to describe whether or to which extent a person finds his or her environment and life circumstances understandable, manageable, and predictable. The SOC-29 has three subscales: Comprehensibility, Manageability, and Meaningfulness. All items were scored on a 7-point Likert scale. High scores should be associated with higher levels of SOC. In our sample, the Cronbach’s *α* for the SOC was 0.88 (*M* = 4.47; SD = 0.75), and alphas in the three subscales were as follows: 0.75 for Comprehensibility (*M* = 3.97; SD = 0.82), 0.76 for Manageability (M = 4.56; SD = 0.90), and 0.79 for Meaningfulness (*M* = 5.03; SD = 0.98).

## Results

We analyzed data with Pearson’s correlation coefficients to assess the relation between Post-Critical Beliefs and SOC scores. The second level of analyses included a series of canonical analyses which we used to demonstrate a relationship between a set of predictor variables (Post-Critical Beliefs) and a set of criterion variables (subcomponents of SOC). In Table 2, we presented descriptive statistics for the PCBS and the SOC-29 scales, separately for women and men, in three age groups.

**Table 2:** Descriptive statistics for women and men in different age groups

Scale	Age 18–30				Age 31–50				Age 51–79			
	Women		Men		Women		Men		Women		Men	
	*M*	SD	*M*	SD	*M*	SD	*M*	SD	*M*	SD	*M*	SD
Orthodoxy	3.90	1.06	3.64	1.08	4.60	0.91	4.38	0.94	5.13	1.01	4.66	1.06
External critique	2.97	0.97	3.27	1.06	3.02	0.84	3.08	0.93	3.22	1.13	3.39	0.97
Relativism	4.10	0.86	4.37	0.91	3.85	0.93	3.91	0.95	3.95	1.12	4.02	0.92
Second Naivetè	4.81	0.83	4.76	0.78	5.05	0.66	4.88	0.75	5.19	0.76	4.91	0.85
Comprehensibility	3.67	0.82	3.96	0.66	3.87	0.87	4.11	0.78	4.09	0.89	4.20	0.81
Manageability	4.54	0.95	4.82	0.71	4.38	0.83	4.68	0.85	4.40	1.04	4.56	0.93
Meaningfulness	5.17	0.95	5.28	0.81	4.96	0.89	5.02	1.08	4.84	1.09	4.98	1.01
SOC	4.38	0.77	4.62	0.61	4.35	0.70	4.56	0.78	4.40	0.85	4.54	0.76

In the PCBS, for Orthodoxy, we noted that the older the participants were, the significantly higher the results we observed. This trend applied to women (*F* = 43.41, *p* < 0.001) as well as men (*F* = 26.29, *p* < 0.001). In addition, proportionally to the age, Second Naiveté (*F* = 7.38, *p* < 0.001) increases in women and Relativism (*F* = 6.73, *p* < 0.01) decreases in men. Within SOC, age differentiated neither women nor men significantly. However, we noted differences in SOC subcomponents. In women, two subcomponents change significantly: Comprehensibility grows (*F* = 6.66, *p* < 0.001) and Meaningfulness decreases (*F* = 3.26, *p* < 0.05). We have not noticed such significant discrepancies in men, but the results tender upwards within Comprehensibility (*F* = 2.54, *p* = 0.080) and downwards for Meaningfulness (*F* = 2.90, *p* = 0.056) and Manageability (*F* = 2.46, *p* = 0.087).

We also noted differences between men and women in individual age groups. In the age group of 18–30 years, in the PCBS, men achieved higher results in External Critique (*t* = −2.18, *p* < 0.05) and Relativism (*t* = −2.24, *p* < 0.05) than women; in the SOC-29, men reported higher results than women in SOC (*t* = −2.47, *p* < 0.05), Comprehensibility (*t* = 2.83, *p* < 0.01), and Manageability (*t* = −2.39, *p* < 0.05). In the age group 31–50 years, we noticed differences only in the SOC-29: men had higher results in SOC (*t* = −1.99, *p* < 0.05), Comprehensibility (*t* = −2.09, *p* < 0.05), and Manageability (*t* = −2.52, *p* < 0.05). In the age group 51–79 years, the differences referred only to the PCBS: in Orthodoxy (*t* = 3.24, *p* < 0.001) and Second Naiveté (*t* = 2.57, *p* < 0.05), we observed higher results in women than in men.

In Table 3, we presented the correlation values between the PCBS and the SOC-29 scale for women and men, separately for each adulthood stage. In the female group, we noted the strongest associations between the PCBS and the SOC-29 in the late adulthood: SOC correlated negatively with the measures assuming the Exclusion of Transcendence (External Critique and Relativism). We also observed negative correlations between External Critique and three SOC subscales. Relativism correlated negatively with Manageability and Meaningfulness. In women in the period of middle adulthood, we observed only one negative correlation between External Critique and Meaningfulness. In early adult women, we found a negative correlation between External Critique and SOC, and Meaningfulness (Table 3).

**Table 3:** Correlation between Post-Critical Beliefs (PCBS) and SOC (SOC-29) for women and men in different age groups

Sex	PCBS	Age 18–30				Age 31–50				Age 51–79			
		Compr	Manag	Meanin	SOC	Compr	Manag	Meanin	SOC	Compr	Manag	Meanin	SOC
Women	ORT	−0.13	−0.07	−0.03	−0.09	−0.04	0.02	0.02	−0.01	−0.05	−0.19	−0.13	−0.14
	EXT	−0.10	−0.17	−0.29**	−0.21*	−0.02	−0.16	−0.26**	−0.16	−0.21*	−0.37***	−0.32***	−0.35***
	REL	−0.14	−0.01	−0.02	−0.06	−0.07	−0.15	−0.17	−0.15	−0.18	−0.30***	−0.30***	−0.31***
	SNA	−0.04	0.03	0.07	0.02	0.03	0.11	0.03	0.07	0.09	0.13	0.06	0.11
Men	ORT	0.06	−0.06	0.01	0.06	0.35***	0.32***	0.33***	0.37***	−0.03	−0.06	0.01	−0.03
	EXT	−0.03	−0.18	−0.26	−0.18**	−0.27**	−0.39***	−0.48***	−0.43***	−0.08	−0.26**	−0.23*	−0.23*
	REL	−0.03	−0.02	−0.01	−0.03	−0.27**	−0.26**	−0.21*	−0.28**	−0.18	−0.22*	−0.07	−0.19
	SNA	−0.04	−0.12	−0.02	−0.07	0.17^	0.26	0.36***	0.30**	−0.13	−0.03	0.12	−0.02

*Compr* comprehensibility, *Manag* manageability, *Meanin* meaningfulness, SOC Sense of Coherence, *ORT* orthodoxy, *EXT* external critique, *REL* relativism, *SNA* Second Naiveté

* *p* < 0.05, ** *p* < 0.01, *** *p* < 0.001

In men, we found most correlations between the PCBS and the SOC-29 at the stage of middle adulthood: measures assuming the Exclusion of Transcendence (External Critique, Relativism) correlated negatively with the SOC-29, whereas measures assuming the Inclusion of Transcendence (Orthodoxy, Second Naiveté) correlated positively with the SOC-29. External Critique and Relativism correlated negatively with SOC and its three components. Orthodoxy and Second Naivetè correlated positively with SOC, in which Orthodoxy also correlated with three, and Second Naivetè with two the SOC-29 subscales (Manageability and Meaningfulness) (Table 3). In early adult men, we found only one negative correlation between External Critique and SOC. In late adult men, External Critique correlated negatively with SOC and its two subcomponents (Manageability and Meaningfulness). Relativism correlated negatively with Manageability (Table 3).

We conducted the canonical correlation analysis to find multidimensional correlations between PBCS and the SOC-29 variable sets in each age group of women and men. As a result, we obtained two significantly correlated canonical variable pairs—one for women aged between 51 and 70, and one for men aged between 31 and 50 (Table 4). The interpretation of canonical variables includes the variables with canonical loadings higher than or equal to 0.40.

**Table 4:** Results of canonical correlation between Post-Critical Beliefs and dimensions of SOC for women aged 51–79 and men aged 31–50

	Canonical variables	
	Women (51–79)	Men (31–50)
*Post*-*critical belief*		
Orthodoxy	−0.03	**−0.67**
External critique	**0.61**	**0.99**
Relativism	−0.35	**0.44**
Second Naivetè	**−0.97**	**−0.81**
*Ad*_*x*_	0.36	0.57
*R*_*x/y*_	0.08	0.18
*R*	0.46	0.56
*R* ^2^	0.22	0.32
*Χ* ^2^	28.65	45.56
*df*	12	12
*p*<	0.01	0.001
*SOC*		
Comprehensibility	**−0.52**	**−0.56**
Manageability	**−0.99**	**−0.77**
Meaningfulness	**−0.76**	**−0.99**
*Ad*_*y*_	0.61	0.64
*R*_*y/x*_	0.13	0.20

Variables with canonical loadings higher than or equal to 0.40 are marked in bold

As shown in Table 4, the canonical correlation coefficient for the value of the variable pair obtained for women aged 51–79 is *R* ^2^ = 0.22, with significance at the level of *p* < 0.01. The results of the second canonical variable (SOC) indicate the variability in their own set more strongly (*Ad*_*y*_ = 0.61) than the results within the first variable (Post-Critical Beliefs) (*Ad*_*x*_ = 0.36). The results of the canonical variable of the PCBS measures explain 13 % (*R*_*y/x*_ = 0.13) of the SOC variance. Eight per cent (*R*_*x/y*_ = 0.08) describes converse dependency, the extent to which SOC explains the diversification of Post-Critical Beliefs. The interpretation direction shows the correlation between SOC and the way of thinking about religion. In the criteria set, three measures of SOC form the canonical variable: Comprehensibility, Manageability, and Meaningfulness. External Critique and Second Naivetè constitute the variable in the predictors’ set (Table 4).

The value of the canonical correlation coefficient for the variable pair in men aged 31–50 is *R* ^2^ = 0.32, *p* < 0.001. The results for the SOC explain the variability in their own set more strongly (*Ad*_*y*_ = 0.64) than the results within the first variable (Post-Critical Beliefs) (Ad_x_ = 0.57). The results of the canonical variable of the PCBS measures explain 20 % (*R*_*y/x*_ = 0.20) of the SOC variance. Eighteen per cent (*R*_*x/y*_ = 0.18) describes converse dependency, the extent to which SOC explains the diversification of Post-Critical Beliefs. The interpretation direction shows the correlation between SOC and the way of thinking about religion. In the criteria set, three measures of SOC form the canonical variable: Comprehensibility, Manageability, and Meaningfulness. External Critique, Orthodoxy, and Second Naivetè constitute the variable in the predictors’ set (Table 4).

## Discussion

Our study examined relations between the PCBS and the SOC scale. We focused on the strength and direction of particular correlations in women and men, in three age groups: early (18–30), middle (31–50), and late adulthood (51–79). We hypothesized that measures which describe the Inclusion of Transcendence (Orthodoxy, Second Naiveté) would correlate positively, and measures which describe the Exclusion of Transcendence (External Critique, Relativism) negatively with SOC. Moreover, we assumed that Meaningfulness will be correlated most strongly with the religious attitudes which include transcendence (Orthodoxy, Second Naiveté) in comparison with other SOC subcomponents. Next, we hypothesized that the salutogenic function of religion may show diversification, depending on age and gender.

1. The first surprising result is that we observed different correlation patterns in women and men in individual age groups. We noted the majority of associations in the late adult women and middle adult men. Therefore, we suppose that the salutogenic function of religiosity may be correlated with gender and age.
2. We obtained another interesting result on the basis of the canonical analysis. It revealed the characteristics of the salutogenic function of religion in groups with the highest number of correlations: in women aged 51–79 and in men aged 31–50. In men, the SOC predictor is the measure of Inclusion versus Exclusion of Transcendence, that is, the SOC rises in the measures of Comprehensibility and Manageability as well as in the measure of Meaningfulness, together with the rise in Orthodoxy and Second Naiveté. In women, the increase in SOC happens together with the rise in Second Naiveté, that is, the acceptance of transcendence and its symbolic understanding (see Fig. 1). Therefore, it seems that the integrating factor in men is the fact of being religious itself, whereas in women, it is not only being religious, but also how they understand religiosity—the more symbolic elements there are in their understanding of religion, the more salutogenic function of religion can be found in it.
3. We found it confusing that we observed positive correlations between the dimensions of SOC and Orthodoxy and Second Naiveté only in the male sample, aged 31–50; negative correlations of External Critique and Relativism with SOC subcomponents appeared in all groups, although middle adult men and late adult women report the widest range of relations. In women aged 18–30, only External Critique correlated negatively with Meaningfulness and with the general SOC. In women aged 31–50, this dependency is analogous but it refers only to Meaningfulness and External Critique. In men aged 18–30, two coefficients suggest a negative correlations of External Critique with Meaningfulness and SOC. However, in men aged 51–79, External Critique correlated negatively with Manageability, Meaningfulness, and the general SOC, and Relativism negatively with Manageability. It is difficult to say why positive correlations between the Inclusion of Transcendence (Orthodoxy, Second Naiveté) and SOC are weaker than the negative correlations between the Exclusion of Transcendence (External Critique and Relativism) and SOC. We suppose that the salutogenic function of religiosity is weakly marked in the general population but it can be activated in various stress and difficult life situations contexts. This needs, however, further research with the inclusion of specific samples. A weak range of correlation between Meaningfulness and the PCBS is also contrary to what we expected. Therefore, we need to perceive this variable rather in the construct of SOC than in the context of the category of sense of life.

Making an effort in the interpretation of these relationships, we referred to the concept of the development of *self* (Kegan 1982) and the concept of developmental studies by Havighurst (1981) as well as to the social roles by Selman (1976). Women display more commitment in the family life and the closest community than men, for example, adult daughters play the role of carers of their elderly parents more often than adult sons (Cantor 1983; Himes et al. 1996). Life targets and decisions in women are more often related to the family matters (Nurmi 1992; Rydz and Ramsz 2007). The superiority of the *social self* over the *universalizing self* in women may prevail until late adulthood (Kegan 1980, 1982) and also determine the coherence processes (Fowler 1980, 1987). We suppose that in women till the late adulthood, the SOC is shaped with reference to complex social constructs more often than to universal matters. Therefore, they “discover” the integrating function of religiosity later (cf. Brzezińska 2000). In men, however, faster development of *universalizing self* and less importance attached to the *social self* result in faster disclosure of the salutogenic function of religiosity than in women.

Some discussions regarding sex and gender differences in religiousness have focused on the role of connected versus separate knowing (cf. Simpson et al. 2008). According to Belenky et al. (1986), separated knowers take an impersonal stance since they rigorously exclude personal beliefs and feelings, while connected knowers emphasize feelings and use empathy and listening to try to understand others. Ozorak (1996) found that “women conceptualize religion in terms of relationship rather than individuation” (p. 23) and noted that women tended to prefer a relational interaction with God as opposed to a more distant participation characterized by individuation (e.g. knowing through reason, viewing God as a distant judge). Thus, religious thinking in women is marked by a stronger bond to the life context than in men, who have more abstractive speculations in their thinking, independently of the context (cf. Francis 1997; Walesa 2003; Pelham et al. 2005). This may partially explain the differences within the specifics of the salutogenic function of religion between women and men. In men, the acceptance of transcendence is the source of SOC, that is, the fact of being religious. In women, however, its source is in the acceptance of the religious system of meanings, connected with its symbolic interpretation. This, in turn, may mean that, in women, religiosity has the salutogenic function as far as they interpret them in the perspective of personal meanings and of life context.

We have to acknowledge certain limitations of our study. In particular, our study does not allow for causal interpretations because of its cross-sectional nature. This is, however, a common limitation of cross-sectional data so that a longitudinal study is needed.

## Conclusion

The study results are inconsistent in terms of the empirical research on the relationships between religiosity and mental health (Gartner et al. 1991). Some authors suggested that religious commitment was associated with better health outcomes (Mueller et al. 2001). Other researchers believed that the postulated relations between religion and health were weak and unconvincing (Sloan and Bagiella 2002). Moreover, many researchers described religiosity as a unidimensional construct. Studies which included multidimensional religiosity concepts were limited to the differentiation between intrinsic and extrinsic religiosity, criticized on both conceptual and psychometric grounds (Kirkpatrick and Hood 1990). The PCBS by Hutsebaut (1996), which operationalizes Wulff’s (1991, 1999) model of approaches to religion, has opened new perspectives for studying religiosity–health’s outcomes relations. In the presented research, we analyzed the relations of Wulff’s approaches with religion and SOC. The results suggest that the salutogenic function of religiosity is related to age and gender—among women, it is most strongly marked in late, and among men, in middle adulthood. In men, the increase in SOC happens together with the rise in the Inclusion of Transcendence, while in women, with the rise in the Inclusion of Transcendence and symbolical understanding of religiosity.
